# Retraction Note: Zebrafish Noxa promotes mitosis in early embryonic development and regulates apoptosis in subsequent embryogenesis

**DOI:** 10.1038/s41419-024-07119-1

**Published:** 2024-10-02

**Authors:** J-X Zhong, L Zhou, Z Li, Y Wang, J-F Gui

**Affiliations:** State Key Laboratory of Freshwater Ecology and Biotechnology, Institute of Hydrobiology, Chinese Academy of Sciences, Graduate University of the Chinese Academy of Sciences, Wuhan, China

Retraction Note to: *Cell Death & Disease* 10.1038/cdd.2014.22, published online 7 March 2014

The Editors-in-Chief have retracted this article at the request of the authors. Duplicated images were noted in figures 3, 6 and & 7 and the authors were unable to locate their original data. The authors have therefore lost confidence in the veracity of the work presented in this article.

None of the authors have responded to any correspondence from the editor about this retraction notice.

